# The relationship among restless legs syndrome (Willis–Ekbom Disease), hypertension, cardiovascular disease, and cerebrovascular disease

**DOI:** 10.1007/s00415-013-7065-1

**Published:** 2013-08-21

**Authors:** Luigi Ferini-Strambi, Arthur S. Walters, Domenic Sica

**Affiliations:** 1 Sleep Disorders Center, Vita-Salute San Raffaele University, Milan, Italy; 2 Department of Neurology, Vanderbilt University School of Medicine, Nashville, TN USA; 3 Virginia Commonwealth University Health System, 1101 East Marshall Street, Sanger Hall, Room 8-062, Richmond, VA USA

**Keywords:** Restless legs syndrome, Prehypertension, Cardiovascular disease, Stroke, Vascular disease, Sleep disturbance

## Abstract

Untreated sleep disorders may contribute to secondary causes of uncontrolled hypertension, cardiovascular disease (CVD), and stroke. Restless legs syndrome, or Willis–Ekbom Disease (RLS/WED), is a common sensorimotor disorder with a circadian rhythmicity defined by an uncontrollable urge to move the legs that worsens during periods of inactivity or at rest in the evening, often resulting in sleep disruptions. Sleep disorders such as insomnia and obstructive sleep apnea (OSA) are established risk factors for increased risk of hypertension and vascular diseases. This literature review outlines the lessons learned from studies demonstrating insomnia and OSA as risk factors for hypertension and vascular diseases to support the epidemiologic and physiologic evidence suggesting a similar increase in hypertension and vascular disease risk due to RLS. Understanding the relationships between RLS and hypertension, CVD, and stroke has important implications for reducing the risks associated with these diseases.

## Introduction

The relationship among hypertension, cardiovascular disease (CVD), and stroke is well established [24]. Hypertension, including higher pulse pressure [systolic (SBP) minus diastolic blood pressure (DBP)], is a major risk factor for myocardial infarction (MI), stroke, heart failure, and renal failure [70, 88]. Increases in SBP and DBP are predictive of long-term risks for chronic heart disease and CVD and result in a progressive and linear increase in the risk of death from heart disease and stroke [24, 83]. Prehypertension is a more recent disease classification and increases the relative risk for CVD, including a 3.5-fold elevated risk for MI and a 1.7-fold increased risk for coronary artery disease (CAD) [100]. Prehypertension was defined in the 7th report of the Joint National Committee on Prevention, Detection, Evaluation and Treatment of High Blood Pressure (JNC-7) as systolic pressures of 120–139 mmHg and diastolic pressures of 80–89 mmHg [24]. A recent meta-analysis of cross-sectional and longitudinal studies reported that prehypertension has an estimated overall prevalence of 36 % and is higher in men (40 %) than in women (33 %) [48]. The prevalence of prehypertension remains constant for subjects aged <50 years and declines with age, presumably as older subjects are diagnosed with hypertension [49].

Many factors contribute to the development of prehypertension, hypertension, and CVD, including age, gender, race, and lifestyle (i.e., obesity, level of physical activity) [145]. Although the percentages of patients whose hypertension is controlled has been increasing, recent studies suggest that 53 % of patients with hypertension do not achieve therapeutic goals [23, 35, 88]. Failing to reach therapeutic goals may be caused by failure to regularly and effectively monitor BP, poor patient adherence to medication regimens, or from failure to recognize secondary causes of hypertension resulting in treatment-resistant hypertension [21, 24, 96].

Untreated sleep disorders such as insomnia, obstructive sleep apnea (OSA), and restless legs syndrome (Willis–Ekbom Disease; RLS/WED) with periodic leg movements during sleep (PLMS) may contribute, at least in part, to secondary causes of uncontrolled hypertension and CVD [20, 65, 66, 69, 127]. For example, Lavie and Hoffstein [69] discovered that patients with poorly controlled hypertension received significantly more antihypertensive medications (*p* = 0.0001) and had significantly higher episodes of OSA per hour (*p* < 0.0005) compared to patients with controlled BP. In addition, increasing OSA severity was a significant predictor of ineffective BP control, suggesting that OSA severity may contribute to the failure of an antihypertensive regimen [69]. Some sleep disorders, particularly RLS and PLMS, often go unrecognized until symptoms of the disorder severely affect patients’ quality of life and daytime functioning. Thus, in predisposed individuals, undiagnosed or untreated sleep disorders such as RLS may provide a partial explanation as to why some patients are unable to achieve therapeutic goals for hypertension [127, 137].

The purpose of this review is to examine the epidemiologic and pathophysiologic evidence for the association between RLS and PLMS with hypertension and CVD. This is presented in the context of discussing what has been learned from insomnia and OSA studies and applying these lessons to the current knowledge in patients with RLS/PLMS. An initial PubMed search was completed from January 1, 2000 to February 1, 2012 using the following terms or combination of terms: restless legs syndrome, periodic leg movements, insomnia, sleep disorders, obstructive sleep apnea, hypertension, blood pressure, sympathetic, prehypertension, cardiovascular (CV), congestive heart failure (CHF), inflammation, stroke, cortisol, hormone, and epidemiology. The search strategy was limited to studies in humans and published in the English language. Additional studies were identified in the bibliographies from the retrieved articles and more recently published papers (since February 2012) have been identified for inclusion from the authors.

## Defining sleep disorders

The sleep disorders discussed in this review include insomnia, OSA, RLS, and PLMS. Patients with insomnia describe symptoms such as an inability to initiate and/or maintain sleep, repeated awakenings, early morning awakenings, a lack of restorative sleep, daytime fatigue, concentration difficulties, and other mental and physical sequelae such as anxiety and depression, and pain [25]. OSA is characterized by the repetitive interruption of ventilation for ≥10 s caused by collapse of the upper airway during sleep, a decrease in oxygen saturation, arousals from sleep, and symptoms of excessive daytime sleepiness [77]. A diagnosis of OSA is often made using polysomnography (PSG) in a sleep lab. PSG is a diagnostic method that simultaneously measures several physiologic variables, including sleep stages using electroencephalograms, electromyograms, and electrooculograms, respiration, and snoring [114]. Disruptions in airflow also can be measured using positive pressure ventilation, where a reduction in inspiratory airflow is indicative of increased upper airway resistance [17]. OSA severity is measured by the apnea/hypopnea index (AHI) defined as the frequency of apneas or hypopneas per hour of sleep. A diagnosis of OSA is typically considered with an AHI of >5 [114]. Thus, an AHI <5 would be considered normal; an AHI ≥30 events per hour would be considered severe.

Restless legs syndrome is a common sensorimotor disorder with an estimated prevalence of 7.3 % in the general population, with women reporting the disease about twice as frequently as men [4]. Because of the lack of physical hallmarks in RLS, a diagnosis is made clinically using four essential criteria established by the International Restless Legs Syndrome Study Group (IRLSSG) [5]. These criteria are founded on the uncontrollable urge to move the legs (or other appendages in more severe cases) that begin or worsen at rest or inactivity, particularly at night, and are only relieved by movement [5]. Recent additions to these criteria by the IRLSSG require the exclusion of mimics, which are conditions that sometimes meet all four criteria for RLS but are not consistent with RLS. Some of these conditions are leg cramps, positional discomfort, leg edema, arthritis, and anxiety [58]. PLMS are present in 85–95 % of patients with RLS and are a diagnosis distinct from RLS [103]. PLMS are diagnosed using PSG during a sleep study using surface electromyographic electrodes to record leg movement or leg muscle (i.e., commonly the anterior tibialis muscles) activation [33]. PLMS are a repetitive series of movements recurring at 5–90-s intervals with each individual movement lasting 0.5–10 s in duration [33]. The movements are frequently associated with arousals from sleep, involve repetitive extensions of the big toe and dorsiflexions of the ankle occasionally involving the knee and hip, and are characterized by involuntary spasms or jerks [33, 94, 127]. OSA is a likely comorbidity with PLMS [1].

## Circadian rhythmicity

Early studies demonstrated the circadian patterns associated with RLS and PLMS [53]. The intensity of the sensory discomfort and motor restlessness with RLS reaches a peak around midnight and a trough around 9:00 am [53, 118]. Dopamine (DA), an important neurotransmitter in RLS and PLMS, shows a circadian rhythmicity with levels being lowest at night [26]. Many aspects of CV function also reflect a circadian rhythmicity and vary during the sleep-wake cycle. For example, mean SBP and DBP readings can be on average as much as 10–20 % lower during sleep than daytime mean values, partially because of decreased sympathetic output [20, 98, 102]. Many other components of CV control also are modulated by the circadian system, including sympathetic activity, cortisol secretion, cardiac vagal modulation, and heart rate [105]. Circadian alterations in heart rate variability have been shown to occur in patients with OSA [86]. Thus, it is logical to hypothesize that disorders that affect the sleep-wake cycle, such as RLS/PLMS, also will influence CV variables.

## The relationship between sleep disturbances, hypertension, and vascular diseases

Much of what we know about the relationship between sleep disturbances, such as those incurred by patients with RLS and PLMS, and the increased risk for hypertension and vascular diseases comes from clinical investigations into such risks in patients with insomnia and OSA. Disturbances in sleep onset, sleep maintenance, and total sleep time are reported by as many as 85 % of patients with RLS with nearly a third of patients with RLS reporting severe sleep disturbances [4, 34, 56]. Because PLMS often accompany RLS and their occurrence frequently results in arousals from sleep, PLMS can exacerbate sleep disturbances [33, 56]. Therefore, an examination of the associations between hypertension and CVD with insomnia and OSA may provide context to similar risks related to RLS and PLMS and may help in the understanding of where CV risks arise in the patients who suffer from RLS and PLMS.

Several studies have suggested that shortened sleep and insomnia may increase the incidence and risk of hypertension and CVD. Prospective and epidemiologic database studies have shown that subjects sleeping ≤5 h per night had up to a 32 % greater likelihood of being diagnosed with hypertension than subjects sleeping longer [42, 44]. Shortened sleep also has been associated with increased risk for CVD and coronary heart disease (CHD). The Monitoring Project on Risk Factors and Chronic Diseases in the Netherlands (MORGEN) study reported that subjects sleeping ≤6 h per night had a 15 % higher risk for total CVD [hazard ratio (HR) 1.15; 95 % CI 1.00–1.32] and a 23 % higher risk (HR 1.23; 95 % CI 1.04–1.45) for CHD compared to subjects sleeping 7–8 h. In addition, short sleep duration and poor sleep quality lead to a 63 % (HR 1.63; 95 % CI 1.21–2.19) and 79 % higher risk (HR 1.79; 95 % CI 1.24–2.58) for total CVD and CHD incidence, respectively, compared to normal sleep duration and quality [54]. It should be noted, however, that the MORGEN study only included a single self-reported item to measure sleep duration and quality. Consequently, sleep duration may have been more representative of time spent in bed rather than actual physiologic sleep as could have been measured by PSG. Finally, patients with insomnia have a blunted nocturnal BP dipping response that may increase the risk of higher BP, CV risk, and target organ damage [31]. In a prospective case–control study in subjects with chronic primary insomnia, Lanfranchi et al. [67] observed significantly higher nighttime SBP (*p* < 0.01), and day and nighttime DBP (*p* = 0.02 and *p* = 0.01) in subjects with insomnia versus those without insomnia. Furthermore, significantly less day to nighttime SBP dipping was observed in the subjects with insomnia (−8 %) versus controls (−15 %; *p* = 0.01), but day to nighttime DBP dipping did not statistically differ between the two groups of subjects [67]. Taken together, these studies suggest that short sleep duration and poor sleep quality may increase the risk for hypertension and CVD.

Obstructive sleep apnea is often comorbid to RLS and PLMS and is established as a significant cause of sleep disturbance and risk factor for hypertension, CVD, and stroke [9, 77, 87]. The Wisconsin Sleep Cohort Study showed that the odds for hypertension increase from 1.42 (95 % CI 1.13–1.78) in patients with an AHI of fewer than five events per hour at baseline to 2.89 (95 % CI 1.46–5.64) in subjects with an AHI of ≥15 events per hour compared to subjects without sleep-disordered breathing [95]. Similar associations between sleep-disordered breathing and increased odds and prevalence of hypertension have been reported in population studies and in the Sleep Heart Health Study [68, 144]. In addition to hypertension, OSA has been reported to increase the risk for atrial fibrillation and CV-related and all-cause mortality [43, 99].

Three key hypotheses have been put forth to explain the influence of OSA on overall CV function, including increased sympathetic drive associated with apneas, chemoreceptor-mediated mechanisms of chronic hypoxia, and changes in cellular mechanisms in response to chronic hypoxia. As may be expected with higher sympathetic drive, patients with OSA do not show reductions in nocturnal BP [113]. In addition, waking sympathetic nerve burst frequency is significantly higher in patients with OSA than in control subjects [113]. Thus, heightened sympathetic activation may be one mechanism by which OSA contributes to the development of a more fixed form of hypertension and subsequent CVD.

## The evidence for an association between RLS/PLMS and hypertension, CVD, and stroke

Prospective and cross-sectional epidemiologic studies, and observational and case studies have examined the potential relationship between RLS/WED and PLMS, and hypertension and CVD or cerebrovascular disease. The sections that follow discuss the studies in detail.

### Prospective epidemiologic studies

Patients with RLS have been reported to have a higher risk of stroke and heart disease than those without the disorder. In a 10-year, prospective, population cohort study examining the effects of sleep disturbance on stroke incidence and heart disease in 1,986 older men, RLS was associated with a 67 % increase in the relative odds for stroke compared to subjects without RLS (adjusted relative odds = 1.67; 95 % CI 1.07–2.60; *p* = 0.024) [37]. The men with RLS also had 24 % higher relative odds (adjusted relative odds = 1.24; 95 % CI 0.89–1.74) of an incident ischemic heart disease event versus patients without RLS, but this difference was not statistically significant. No relationships between BP and RLS or other sleep disturbances were observed [37].

The Nurses’ Health study prospectively examined the effect of RLS disease duration on the risk of CHD in 70,977 women. The women were asked if they had ever received a physician-based diagnosis of RLS. Only among the women with longer RLS duration was a significant relationship between RLS and CHD observed. The women with RLS for ≥3 years had higher HRs for CHD (1.72; 95 % CI 1.09–2.73; *p* = 0.03), nonfatal MI (1.80; 95 % CI 1.07–3.01; *p* = 0.045), and fatal CHD (1.49; 95 % CI 0.55–4.04) compared to women without RLS [74]. Sensitivity analysis revealed similar results for the women with RLS ≥3 years versus those without RLS after adjusting for history of diabetes or arthritis (HR 1.94; 95 % CI 1.11–3.37) and snoring (HR 1.80; 95 % CI 1.09–2.97). A higher frequency of women with RLS reported snoring than those without RLS (25.5 vs 18.6 %, respectively; *p* < 0.05) and using antidepressant medications (27.9 vs 10.7 %; *p* < 0.05). Although these investigators adjusted for snoring, there is no indication that a diagnosis of sleep disordered breathing or OSA was considered further in their analysis. In addition, many antidepressants are known to cause or exacerbate RLS symptoms and may have contributed to the RLS. Given these limitations, however, the number of subjects included in this study adds strength to the overall results of a possible relationship between RLS and CHD.

Winter et al. [143] examined the relationship between RLS and incident CVD in subjects from the Women’s Health Study (WHS; *n* = 3,487 women) and the Physicians’ Health Study (PHS; *n* = 1,373 men). Subjects were screened for RLS using the IRLSSG diagnostic criteria. The women (WHS) were followed for a mean of 6.0 years and the men (PHS) for a mean of 7.3 years. Although the percentage of subjects reporting hypertension at baseline was high in both studies, more subjects with RLS reported a history of hypertension than those without RLS (WHS: 50.4 vs 46.7 %, respectively; *p* <0.01; PHS: 51.2 vs 48.3 %; *p* = 0.04). In the WHS, women with RLS had an age-adjusted increase in coronary revascularization compared to the women without RLS (HR 1.42; 95 % CI 1.10–1.82). After adjustment for vascular risk factors, however, this association was not significant. Contrary to the findings from Li et al. [74], the women with RLS did not have a significantly increased risk for major CVD (HR 1.15; 95 % CI 0.88–1.50), MI (1.01; 95 % CI 0.65–1.57), stroke (1.29; 95 % CI 0.91–1.82), or CVD death (1.11; 95 % CI 0.55–2.25). In the PHS, RLS at baseline was not associated with an elevated risk in any of the CV events analyzed in the age-adjusted or multivariable models, including major CV events, stroke, MI, revascularization, or CVD death [143]. It is possible the self-reported symptoms of RLS by these subjects may have been misinterpreted as conditions that mimic RLS. The exclusion of comorbidities that may mimic RLS in the sensitivity analysis would have corrected for this. It also is unclear whether the severity of RLS or the presence of PLMS was assessed in the sample population. In addition, this study did not control for the duration of RLS as was done in the study by Li et al. [74] showing a positive association of RLS to CVD.

Szentkiralyi et al. [115] evaluated the relationship between CV risk factors and vascular diseases and incident RLS using the results from two prospective studies: the Dortmund Health Study (DHS; *n* = 1,312) and the Study of Health in Pomerania (SHIP; *n* = 4,308) over a mean follow-up of 2.1 and 5.0 years, respectively. Incident RLS was defined as an absence of the disorder at baseline versus a diagnosis of RLS at the follow-up visit. In both the DHS and SHIP studies, the presence of RLS at baseline was not associated with increased incident hypertension. When adjusting for age and gender, the OR for incident hypertension in the subjects with baseline RLS was 0.70 (95 % CI 0.24–2.03; *p* = 0.51). Interestingly, the OR for incident stroke in the DHS subjects with baseline RLS was a nonsignificant 2.46 (95 % CI 0.51–11.93; *p* = 0.27); however, only 12 incident cases were included in this calculation. Conversely, in SHIP, a history of hypertension, MI, or stroke was significantly related to incident RLS when adjusting for age and gender, suggesting that the presence of these CVD risk factors predict the development of RLS [115]. Neither the DHS or SHIP studies evaluated the duration of RLS in the subjects. It also is possible that the follow-up periods of 2.1 and 5.0 years for the DHS and SHIP studies, respectively, were not long enough to detect such relationships. Therefore, although the CV factors predicted the onset of RLS rather than the reverse, these results further suggest a relationship between RLS and CVD albeit in the opposite direction as that reported in the Elwood et al. and Li et al. studies [37, 74].

In terms of the influence of RLS on mortality risk, there have been conflicting reports. In a prospective analysis of data from 18,425 men followed for up to 8 years in the Health Professionals Follow-up Study, Li et al. [75] reported that men with RLS had a higher risk of mortality when also diagnosed with hypertension (HR 1.61; 95 % CI 1.32–1.95) or CVD (HR 1.68; 95 % CI 1.27–2.22). In both cases these risks were higher than when either the hypertension, CVD, or RLS occurred alone. In an age-related analysis, the risk of total mortality increased with the frequency of RLS symptoms from 1 to 14 times per month (HR 1.33; 95 % CI 1.08–1.64) to >15 times per month (HR 1.46; 95 % CI 1.17–1.83; *p* trend <0.0001) [75]. Thus, the results from these analyses suggest that RLS increases the risk of CVD, CHD, and stroke and that the risk may increase with the increasing severity of RLS or the frequency of RLS symptoms. In contrast, a study of four prospective cohort studies in patients with RLS by Szentkiralyi et al. [116], which included patients with hypertension (overall 49.2 % of patients) and CVD (overall 1.4 %) at baseline, reported no relationship between RLS and all-cause mortality [116]. Their analysis included data from the German DHS (*n* = 1,299) and SHIP (*n* = 4,291) studies and the WHS (*n* = 31,370), and PHS (*n* = 22,926) US studies. At baseline among the four studies, the prevalence of RLS ranged from 7.4 to 11.9 % with the women from the WHS having the highest prevalence of RLS at baseline. In addition, the weekly frequency of RLS symptoms or the duration of the disease did not increase the mortality hazard [116]. Differences in these results compared to those of Li et al. [75] may be attributable to differences in study populations as well as the exclusion of potential confounding variables such as diabetes and arthritis in the analysis.

### Cross-sectional epidemiologic studies

In addition to the prospective epidemiologic and observational studies discussed above, a number of cross-sectional epidemiologic studies have examined the potential association between RLS/PLMS and hypertension and CVD. There have been 20 previous cross-sectional epidemiologic studies that have looked at the relationship between RLS and hypertension, heart disease, and stroke. Of these 20 studies, 15 suggested an increased risk of hypertension, CVD, CAD, cerebrovascular disease, or heart disease in patients with RLS/PLMS [2, 10, 14, 16, 40, 61, 71, 78, 84, 87, 97, 121, 132, 136, 137]. Five cross-sectional epidemiologic studies reported no associated or a reduced risk of hypertension and CVD compared to patients without RLS [30, 55, 101, 141, 142]. As with the prospective and observational studies, the differences in the outcomes from the cross-sectional epidemiologic studies may be attributed to differences in sample populations, diagnostic criteria for RLS, and the consideration of disease duration and severity.

### Observational studies

A series of observational studies have reported a greater prevalence of cerebrovascular events in patients with RLS compared to control subjects. Walters et al. [126] compared the MRI scans from patients with RLS to those of control subjects without RLS. When controlled for age, gender, and comorbidities, the likelihood for any stroke was higher in patients with RLS compared to controls, but was not significant (OR 2.46; 95 % CI 0.97–6.28; *p* = 0.06). Patients with RLS also had a nonsignificant higher incidence of silent infarctions (19.2 % of patients vs 12.0 % of controls), large subcortical lesions (42.3 vs 36.9 %, respectively) and higher mean cerebral atrophy scores (8.96 vs 8.58, respectively) compared to age-matched controls without RLS. As indicated by the MRI, patients with RLS had slightly more affected subcortical volume and more cerebral atrophy than the non-RLS controls [126]. A more recent study by Boulos et al. [19] extends these findings by reporting a strong positive correlation between the number of leg movements per hour with the presence of white mater hyperintensities (*r* = 0.70; *p* < 0.01). Caused by chronic hypoperfusion and plasma leakage into the white matter due to small vessel disease, white matter hyperintensities are common in patients with CV risk factors and cerebrovascular disease [32].

In some patients, however, RLS and PLMS may arise as a consequence of a stroke. Lee and coworkers [73] used conventional MRI to evaluate patients experiencing RLS symptoms following an ischemic stroke. Of the 137 patients enrolled in the study, 17 (12.4 %) were diagnosed with RLS after the stroke. Most patients who developed RLS had infarcts in subcortical regions, including the basal ganglia/corona radiate, pontine region, thalamus, and internal capsule; only one in 54 patients (1.9 %) who had cortical lesions with or without subcortical involvement were diagnosed with RLS. The symptoms of RLS appeared a mean of 1.8 days (range 1–4 days) following the stroke [73]. The results from the study by Lee et al. [73] are consistent with those from three prospective studies showing that symptoms of RLS and PLMS may arise as a consequence of a stroke. Benbir et al. [13] reported that 54 % of patients (19/35 patients) had a PLMS index >15 after an acute supratentorial ischemic stroke versus 17.1 % of healthy control subjects [13]. In addition, in a separate study in patients following a subarachnoid hemorrhage, 25 % of the patients (5/20 total patients) were subsequently diagnosed with symptoms consistent with RLS/PLMS [106]. In both these studies, none of the patients had a previous history of PLMS or RLS [13, 106]. In a study by Medeiros et al. (*n* = 96) [80], although many of the patients reported having previously undiagnosed RLS symptoms prior to the stroke, symptoms of the disorder worsened after the stroke. The patients with RLS presented with more severe stroke symptoms at baseline, and stroke outcome was significantly worse in the patients with RLS after 3 (*p* = 0.004) and 12 months (*p* = 0.001) compared to the patients without RLS [80]. In addition to worsening RLS symptoms, Coelho et al. [27] showed that patients with a history of stroke also have a greater prevalence of PLMS (stroke: 47.5 %; controls: 12.5 %) and significantly higher mean PLMS index (stroke: 11.7 ± 3.4; controls: 1.9 ± 0.7; *p* = 0.006) than control subjects [27]. The PLMS index was similar between the two patient groups. More patients with a stroke had RLS than the controls (30 vs 17.5 % of patients, respectively), but this difference was not significant. It is tempting to speculate that the lesions in the cortical and subcortical sensorimotor regions following the stroke may have influenced the development of RLS and PLMS in these patients. However, not all patients with such infarcts developed symptoms consistent with RLS or PLMS. This, perhaps, supports the hypothesis that the development of RLS and PLMS arises from a complex interplay of several distinct pathways. More importantly, the appearance or worsening of symptoms consistent with RLS and PLMS may serve as an indication of stroke severity or poorer stroke outcome and should be monitored in patients experiencing a stroke.

Most of the studies on sleep disturbances, including RLS/PLMS, have been reported in adult populations. Thus, a study by Wing et al. [134] provides important observations on the effects of PLMS on hypertension in 314 children with sleep problems (mean [SD] age 10.4 [1.7] years; 62.4 % boys). These investigators compared the changes in nocturnal SBP and DBP between children with PLMS to children without PLMS (controls). The presence or severity of RLS was not assessed. The children with PLMS were at significantly higher risk for nocturnal systolic hypertension (adjusted OR 6.25; 95 % CI 1.87, 20.88; *p* < 0.05), diastolic hypertension (adjusted OR 4.83; 95 % CI 1.66, 14.07; *p* < 0.05), and both systolic and diastolic hypertension (adjusted OR 18.49; 95 % CI 4.60, 74.27; *p* < 0.05) compared to the children without PLMS. Further, there tended to be a higher proportion of diastolic non-dippers in the children with PLMS (adjusted OR 3.04; 95 % CI 0.95, 9.77; *p* = 0.06). The risks for daytime systolic and diastolic hypertension also were higher in the children with PLMS [(systolic adjusted OR 2.44; 95 % CI 0.48, 12.48; diastolic adjusted OR 1.57; 95 % CI 0.18, 13.9), respectively], but these did not reach statistical significance [134]. These results are important because pediatric hypertension, including prehypertension, is often carried into adulthood. Identifying PLMS as a possible contributor towards the development or presence of hypertension in childhood may prevent the development of future CVD in at least some individuals.

Because they occur in 85–95 % of patients with RLS [103], it is important to consider the effects on PLMS on the risk for CVD. In a study of 2,911 men aged ≥65 years over a 4-year period, Koo and colleagues [66] reported increasing CVD odds with increasing frequency of PLMS and arousals. In patients experiencing arousals in conjunction with PLMS (the PLM arousal index [PLMAI]), those with one to fewer than five arousals per hour had a 19 % higher incidence of incident CVD, whereas patients with ≥5 PLMS-related arousals per hour had a 26 % higher incidence of incident CVD (HR 1.26; 95 % CI 1.01–1.57; *p* trend 0.0402) [66]. Interestingly, although patients with 5–30 PLMS per hour had a higher likelihood of incident hypertension, patients with ≥30 PLMS per hour did not. The authors reasoned that the lack of an association between incident hypertension and ≥30 PLMS per hour may have been caused by the high prevalence of hypertension in this cohort [66]. It is not clear whether the PLMS were in relation to RLS in these patients or the severity of the RLS. Further analysis of this patient sample revealed a significant increase in unadjusted odds of atrial fibrillation in men with ≥30 PLMS per hour compared to the men with <5 PLMS per hour (OR 1.57; 95 % CI 1.03–2.39) [65]. Thus, these data suggest that the frequency of PLMS with or without arousals is a potential predictor of incident CVD, including atrial fibrillation, in older men, whereas the relationship between PLMS and incident hypertension is less clear [65, 66].

Patients with CHF also have a high prevalence of PLMS. In a study by Hanly and Zuberi-Khokhar [52], 52 % of the patients with CHF had >25 PLMS per hour and 33 % had >50 PLMS per hour of sleep; PLMS were present in about 10 % of healthy controls, which is consistent with the overall PLMS prevalence in the general population [107]. These results, however, are based on the outcome of 23 subjects. This study did not assess the presence of RLS in the patients. Two other studies also observed 20 % prevalence rates for PLMS in patients with CHF [59, 111]. In some of the patients, the PLMS were associated with cardiac accelerations and a significant increase in heart rate in the absence of electroencephalographic activation, whereas in other patients, PLMS resulted in a mildly increased number of arousals (3.4 ± 2 per hour) [59].

With all these studies, the direction of the causality between PLMS and CHF cannot be ascertained. However, a case study report by Hanly and Zuberi [51] described a male patient with CHF who received a heart transplant following an extensive MI. Polysomnography performed 3 months prior to the transplantation revealed as many as 158 PLMS per hour, of which 32 % (51/158) were accompanied by arousals. The patient also had an AHI of 39. After the transplantation, the PLMS improved to 12 per hour and arousals to 3 per hour, with an AHI of 5 per hour following receipt of the new heart [51]. This suggests that reductions in CV function, such as reduced hemodynamics brought on by cardiac dysfunction, may increase the risk of PLMS in a subset of patients. Conversely, Javaheri et al. [60] reported an increase in the prevalence of PLMS and AHI in patients who underwent heart transplantation. Five months after the transplantation, 31 % of the 45 transplant recipients had ≥15 PLMS per hour with an average of 55 per hour and a PLMS index ranging from 16 to 142 per hour. Thirty-six percent of the heart recipients had an apnea index of >15 episodes of apnea an hour with an average of 50 episodes per hour. These results led the authors to argue in favor of performing PSG monitoring on patients receiving heart transplants [60].

### Case studies

A few case studies have been published reporting the presence of RLS and/or PLMS in patients with hypertension or cerebrovascular disease. Of the eight published case studies, one reported increases in systemic BP related to PLMS [3], one reported symptoms consistent with RLS preceding the event and which worsened after the ischemic event [7], and six reported the development of RLS/PLMS following the stroke [62–64, 72, 108, 122]. Although these studies report that the symptoms of RLS/PLMS began after the stroke, many of these patients had an pre-existing history of hypertension or CVD. In addition, the development of RLS or PLMS symptoms was independent of the location of the infarct; however, many of the strokes involved areas of the brain involved in motor control, such as the basal ganglia or pyramidal tracts, or in sensorimotor integration including the thalamus.

In summary, prospective and cross-sectional epidemiologic studies, and observational and case studies provide evidence for an association between RLS and PLMS and the risk for hypertension and CVD and the effects of these movement disorders on mortality. These associations are based, in many instances, on self-reported RLS symptoms and the presence of hypertension and CVD. In some studies, factors associated with CVD, such as MI or stroke, have been posited to lead to increased risk of RLS. Thus, establishing a relationship does not imply causality or the direction of that causality. Whether these increases in statistical risks translate into clinically meaningful elevations in risk, however, are as yet unknown.

The variance in the results may reflect differences in study design, diagnostic criteria for RLS, studied population (general vs clinical), severity of RLS at baseline, comorbidities, and medication behavior. The association between RLS and hypertension or CVD also may be dependent on the severity of the RLS at baseline or the duration of the disease. This was clearly the case in the studies by Li et al. and Koo et al. [66, 74] in which the duration of disease and severity of PLMS led to higher risks of CVD. Given these differences in study design, however, the variability with which the results point to an association between RLS/PLMS and hypertension and more so CVD, illustrates the complexity of the interaction between RLS and CVD in the selected sample populations.

## Additional analyses evaluating the relationship between RLS and hypertension, CVD, and stroke

A recent systematic review [57] comparing the outcomes of several epidemiologic, retrospective, and observational studies examining the relationships between RLS and metabolic dysfunction, sympathoadrenal dysfunction, hypertension, and CVD risk helps to put the results of the studies discussed in this review into context. The prevalence of RLS in subjects with hypertension ranged from 6 to 34 %. Overall, a strong positive association was found between RLS and hypertension with OR ranging from 1.35 to 2.1 [57]. The prevalence of RLS in patients with CVD ranged from 7.7 to 36 %, which is three times the prevalence of RLS observed in the general population without CVD. Most of the selected studies reported a significant positive relationship between RLS and CVD. After applying a more stringent definition of RLS based on the IRLSSG criteria, the OR for RLS and CVD ranged from 2.1 to 2.9 after adjustment for confounding variables. Thus, the analysis supports the hypothesis that RLS may be strongly and positively related to CVD [57].

The results of the systematic review by Innes et al. [57] are consistent with and expand upon a review by Walters and Rye [127] investigating relationships between RLS/PLMS and hypertension and CVD. The review described in detail the results of several studies supporting the association between RLS/PLMS and CHF, hypertension, and stroke as well as BP fluctuations. However, the authors were careful to note that RLS and PLMS have appeared in patients following the onset of acute clinical stroke and, hence, RLS/PLMS could be caused by heart disease and peripheral vascular disease. The evidence provided in this review strengthens the positive findings of the systematic review by Innes et al. [57] by providing greater detail on the relationships between RLS/PLMS and hypertension and CVD.

## Potential mechanisms between RLS/PLMS with hypertension, CVD, and stroke

Whereas epidemiologic studies have examined the relationship between RLS/PLMS and hypertension and CVD, studies examining the underlying physiologic and biochemical mechanisms connecting RLS/PLMS and hypertension CVD may help to explain the processes by which RLS/PLMS may lead to hypertension and CVD, or vice versa, in certain patients.

### Physiologic mechanisms

The processes by which RLS/PLMS may influence the development of hypertension and CVD have been suggested from PSG studies in which PLMS were shown to increase BP and heart rate. In patients with PLMS without RLS, Winkelman showed that PLMS were associated with significant increases in heart rate from baseline, which were higher than those seen in periodic leg movements during waking [135]. This result was based on an analysis of 796 leg movements from 8 patients. Most of the PLMS (73 %) occurred in stage 2 sleep. The increases in heart rate began three cardiac cycles (defined by the RR interval) prior to the movement and rapidly increased to maximum just after the movement [135], a result replicated by later investigators [92, 93]. Pennestri et al. [93] extended these observations to show that a significant SBP and DBP also occurs following PLMS in healthy subjects (i.e., subjects without RLS).

Espinar-Sierra et al. found a proportional relationship between the severity of PSG-measured PLMS and increased daytime hypertension. Patients with grade III hypertension (World Health Organization classification; SBP ≥180 mmHg, DBP ≥110 mmHg [133]) had a significantly higher prevalence of PLMS compared to patients with grade I/II (SBP 140–179 mmHg, DBP 90–109 mmHg [133]) hypertension (36.4 vs 13.0 %, respectively; *p* < 0.02) [39]. Many of the patients in this study (85 %) were taking antihypertensive medications, some of which, in particular calcium channel blockers, may have induced PLMS. Furthermore, in a study of 861 subjects, Billars et al. [18] reported that the likelihood of hypertension increased more than two-fold in subjects with a PLMS index >30 PLMS/h (OR 2.26; 95 % CI 1.28–3.99) independent of age and body mass index. The results of these studies show that the presence of PLMS can increase BP and heart rate during sleep, and the increasing severity of the PLMS may increase the risk of hypertension and possibly subsequent CVD. In patients with RLS, large increases in BP on the order of 20 mmHg are seen at the time of PLMS as documented in the studies of Siddiqui et al. [110] and Pennestri et al. [92]. In Pennestri et al. [92] the magnitude of the BP response to the PLMS was correlated to the duration of the RLS (SBP: r = 0.76, *p* = 0.02; DBP: *r* = 0.77, *p* = 0.02).

**Fig. 1:**
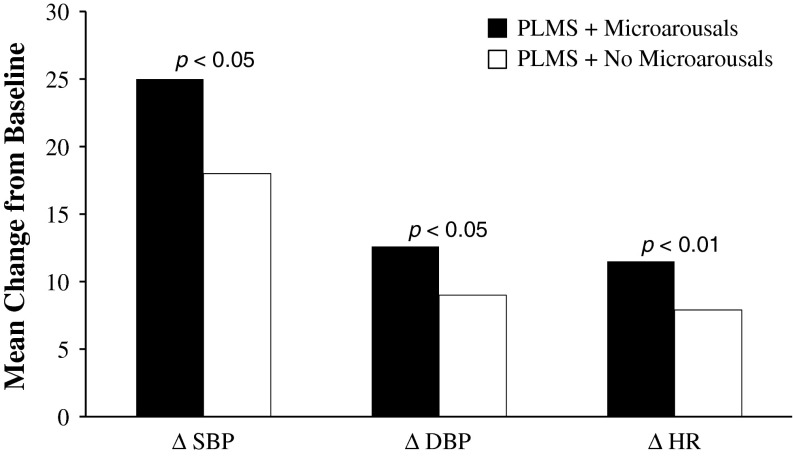
Microarousals in PLMS resulted in significantly higher increases in HR, SBP, and DBP than the PLMS without microarousals. *DBP* diastolic blood pressure, *HR* heart rate, *PLMS* periodic leg movements during sleep, *SBP* systolic blood pressure. Data from Pennestri et al. [92]

Increases in CV variables, such as BP and heart rate, have been shown to be 10 to 35 % higher when associated with EEG-defined microarousals from sleep than without microarousals [36, 92, 110, 135]. Walters and Rye postulated that the increases in BP and heart rate seen in patients with EEG-defined microarousals associated with PLMS were likely due to sympathetic activation [127]. Although Pennestri et al. [92] showed that PLMS with or without arousals from sleep are associated with significant increases from baseline in heart rate, SBP, and DBP (*p* < 0.001 for all), the increases in heart rate, SBP, and DBP were significantly greater with PLMS and microarousals than without microarousals (*p* < 0.05, *p* < 0.05, and *p* < 0.01, respectively) (Fig. 1). The increases in SBP and DBP were strongly associated with microarousal duration (*r* = 0.87, *p* = 0.002; *r* = 0.90, *p* = 0.001, respectively) and increased disease duration (*r* = 0.76 and *r* = 0.77, respectively; *p* = 0.02 for both) [92]. In a similar study in patients with moderate to severe RLS, Siddiqui et al. [110]. also demonstrated that when associated with arousals, PLMS result in significant increases in SBP (mean [SD] maximum rise: 11.2 [8.7] mmHg; *p* < 0.05) and DBP (8.4 [6.4] mmHg; *p* < 0.05) compared to voluntary leg movements. SBP significantly increased when the PLMS occurred without arousals [11.2 (8.7) mmHg; *p* < 0.05] compared to the voluntary leg movements. The voluntary leg movements served as control movements for the PLMS. The patients in this study had a mean index of 10 ± 5 PLMS/h. The maximum rise in SBP occurred 5 s after a PLMS and 6 s after a PLMS-related arousal; increases in DBP occurred 1 s after a PLMS and PLMS arousal. Mean (SD) heart rate due to PLMS with and without arousals increased [4.8 (3.7) and 5.9 (4.6)], respectively), but were not significant compared to the voluntary leg movements. The significant increases in SBP and DBP in relation to PLMS with arousals is consistent with the finding of Pennestri et al. [92]. The authors concluded that the consistent PLMS-related rise in SBP, DBP, and heart rate compared to the voluntary leg movements was indicative of sympathetic activation, which is more pronounced when associated with arousals from sleep [110]. The studies by Pennestri et al. [92] and Siddiqui et al. [110] imply that the PLMS, both with and without arousals, drives the autonomic nervous system from a state of predominant vagal tone during sleep to increased sympathetic drive during such movements. Siddiqui et al. noted several imitations of their study, however, including measuring the respiratory effort related to arousal and that some patients were taking antidepressants that may cause or exacerbate RLS symptoms.

Manconi et al. [79] reported significantly higher differences in heart rate associated with PLMS in patients with RLS versus healthy controls (*p* < 0.05). In the absence of PLMS, no statistical differences in heart rate were observed between patients with RLS and control subjects, suggesting that the heart rate changes associated with PLMS were not caused by primary CV dysfunction [79]. This study involved 23 patients with RLS and 10 control subjects. However, RLS symptom frequency had to be at least 6 months and categorized as severe, and patients with RLS had to be free of any medication at the time of the study [79].

In a more recent study, Pennestri et al. [93] determined the effects of PLMS on heart rate and BP in healthy subjects and to compare the amplitude of the effects of PLMS on SBP, DBP, and heart rate between patients with RLS and healthy controls. Consistent with the above Manconi study [79], PLMS led to significant increases in heart rate, SBP, and DBP compared to baseline (*p* < 0.05) in the healthy subjects; these changes were higher when the PLMS were associated with microarousals. Pennestri et al. [93] extended the findings of Manconi et al. [79] by reporting that the PLMS-related percent increases in SBP, DBP, and heart rate from baseline all were greater in patients with RLS compared to healthy controls. In the absence of microarousals, only the PLMS-related percent changes in heart rate between the patients with RLS and healthy controls achieved significance (*p* = 0.003 for interaction). However, PLMS-related percent increases were significantly greater for heart rate (interaction *p* = 0.02), SBP (interaction *p* = 0.04), and DBP (interaction *p* = 0.02) in the patients with RLS compared to the healthy controls when the PLMS were associated with microarousals [93].

Taken together, the above studies support the hypothesis that RLS/PLMS result in increases in BP and heart rate, which are greater in magnitude when associated with arousals from sleep and establish a potential link between PLMS and daytime hypertension. Thus, the number of EEG-defined arousals from sleep may be a significant predictor of higher SBP, DBP, and heart rate [36]. On the other hand, the rise in BP associated with PLMS not associated with EEG arousals is quite significant [110]. There is considerable evidence in the literature that elevated nocturnal BP alone may be a risk factor for the development of CVD in the general population. Non-dippers (i.e. those subjects whose BP does not drop at night) have more CVD than dippers (i.e. those subjects whose BP does drop at night) in the general population. It is certainly possible that subjects with RLS with nocturnal rises in BP from PLMS may also be at increased risk for CVD similar to non-dippers whether or not they have daytime hypertension. This may be one of the reasons that some of the epidemiologic studies show a relationship between RLS and CVD but not between RLS and daytime hypertension. Interestingly enough, recent studies do show that RLS patients tend to have the non-dipping BP pattern at night compared to normal controls [38]. It is also certainly possible that the nocturnal rise in BP associated with PLMS may serve as a priming factor for the development of daytime hypertension. Although the PLMS appears to be the origin of the increases in BP and heart rate, the fact that PLMS are present in 85–95 % of patients with RLS [103] helps to reconcile some of the variation in epidemiologic data reporting an association between RLS, hypertension and CVD suggesting that an elevated risk of CVD should be considered for the majority of patients with RLS.

Conversely, there is evidence suggesting that the increases in BP and the blunting of nocturnal BP dipping occur independent of PLMS or arousals from sleep. Recently, patients newly diagnosed with essential grade I hypertension were shown to have higher 24 h BP and heart rate and blunted nocturnal BP dipping [47]. In addition, the hypertensive patients were found to have a significantly higher increase in morning BP prior to waking than healthy control subjects. However, the investigators concluded that the PLMS did not contribute to the observed nighttime CV profile [47]. The PLMS index (hypertensive patients: 4 ± 8; controls: 3 ± 6) and the number of PLMS-related arousals (hypertensive patients: 1 ± 4; controls: 1 ± 2) were comparable between the hypertensive patients and controls and considerably less in severity than the studies reporting a positive relationship between PLMS and CV risk [66]. Also, except for slight differences in non-rapid eye movement sleep, the sleep parameters were comparable between the two study groups, including sleep fragmentation. One possible explanation for the difference in PLMS-related hemodynamic outcomes is that the effects of PLMS and PLMS-related arousals on CV variables, including changes in BP and nocturnal BP blunting, are dictated by the severity of the PLMS. That is, there is a threshold where PLMS severity induces pathologic changes in BP. An alternative explanation is likely that PLMS and related arousals do not affect BP in all patients. Further study will likely provide additional clues to provide a greater understanding of these relationships.

### Inflammatory cellular pathways

Although only a few studies with small subject numbers have examined the cellular processes that may be related to an increased the risk of CVD in patients with RLS/PLMS, these studies provide additional clues for a biologic mechanism between RLS and CVD. The association between inflammation and heart disease is well established. For example, elevations in specific inflammatory mediators such as C-reactive protein (CRP) have been identified as risk factors for the development of CVD [24]. The pathways by which inflammatory pathways may stimulate RLS and PLMS, however, are less clear. The results of the studies in patients with RLS are consistent with the observations of similar inflammatory processes in patients with insomnia or OSA.

Trotti et al. [119] reported patients with RLS and elevated CRP had a significantly higher incidence of PLMS than patients with low CRP (*p* = 0.04). The authors also calculated that a single PLM per hour corresponds to a 1.5 % increased risk for elevated CRP [119]. Trotti et al. [120] also reported a modest correlation (*r* = 0.19) between PLMS and elevated CRP values in patients with PLMS >45 per hour (OR 3.56; 95 % CI 1.26–10.03; *p* < 0.02). The number of PLMS per hour were greater in patients with hypertension and cerebrovascular disease than in patients without these comorbidities. After adjusting their model for age, gender, hypertension, inflammation, CRP-lowering medication, and other covariates, PLMS >45 per hour remained a significant predictor of CRP (OR 8.60; 95 % CI 1.23–60.17; *p* < 0.03) [120]. This study did not, however, include a control group completely free of RLS and PLMS or consider the effect OSA on inflammatory values. Nevertheless, the authors posit the results suggest that high PLMS in patients with RLS may contribute to increased systemic inflammation and subsequent CVD.

Lipoprotein-associated phospholipase A2 and highly sensitive-CRP (hs-CRP) are also considered novel inflammatory markers of CV and cerebrovascular events [11]. Both these inflammatory proteins have been shown to increase in relation to the severity of PLMS. In patients with a PLM index ≥15, lipoprotein-associated phospholipase A2 was significantly greater than in patients with a PLM index <15 (206.8 ± 78.1 vs 157.8 ± 56.7 ng/ml; *p* = 0.003). The severity of PLMS was moderately correlated with the protein’s serum concentration (*r* = 0.40). After linear regression analysis, the protein was shown to be a positive predictor of PLM index (*R* ^*2*^ = 0.36), where every 10 ng/ml increase in lipoprotein -associated phospholipase A2 resulted in 1.9 unit increase in PLM index [11]. Similar results were observed for hs-CRP: hs-CRP was significantly higher in the subjects with a PLM index ≥15 versus those with an index of <15 (4.2 ± 3.5 vs 2.4 ± 2.1, respectively; *p* = 0.02). However, only a weak correlation was found between the PLM index and hs-CRP level (*r* = 0.24) [11]. Thus, the results from this study suggest that a high PLM index may be indicative of greater activity of inflammatory markers associated with CVD and cerebrovascular disease.

The results of Trotti et al. [119, 120] and Bekci et al. [11] are consistent with previous studies, suggesting chronic sleep restriction may itself increase inflammatory responses. For example, an increase lymphocyte activation and the production of inflammatory cytokines, including interleukin (IL)-1β, IL-6, and IL-17, as well as CRP and the secretion of tumor necrosis factor-α (TNF-α), has been shown in men sleeping ≤6 h per night [50, 123, 125]. Furthermore, van Leeuwen et al. [123] found that serum CRP significantly increased 145 % from baseline (*p* < 0.05) following 4 h of restricted sleep over a 5-day period and increased by 231 % after sleep recovery versus baseline (*p* < 0.05). The association between CRP and PLMS in the Trotti study also may be the result of disturbed sleep as is common in patients with RLS/PLMS. Thus, the chronic sleep restriction that typically results from RLS/PLMS may serve as one mechanism for the increase in inflammatory activity, thereby contributing to the development of CVD, further supporting a biologic connection between sleep disturbances and CVD development. CRP and hs-CSP have been associated with the development of atherosclerosis and other CV complications through an increase in carotid intima-media thickness [22, 41]. However, patients with RLS, while suspected of having higher levels of these proteins than controls, have been shown to have significantly lower mean maximal intima-media thickness compared to subjects without the sensorimotor disorder (*p* < 0.05) [89]. Whether these cellular processes are the result of RLS/PLMS or a causative factor related to the development of CVD remains to be elucidated.

In their review of the potential inflammatory mechanisms associated with RLS, Weinstock et al. [131] found that 89 % of the conditions associated with RLS also are involved with many immune or inflammatory diseases, and 43 % were related to systemic iron deficiency. Combining the observations of Weinstock et al. with the results from the previously reviewed studies suggests that inflammatory processes are an important underlying factor in the development of RLS. Additional studies examining the relationship between inflammation, CVD, and RLS may help to identify the cellular processes that may connect these two diseases.

### Brain metabolic pathways

As previously discussed, Szentkiralyi et al. [115] proposed that hypertension, MI, or stroke significantly predict the onset of RLS. This possibility may be related to disruptions in brain metabolic pathways that affect the development of RLS. Although its role in the risk for hypertension and CVD has yet to be fully understood, iron deficiency has a well-established role in the development in RLS; suggested mechanisms include impairments to DA neurotransmission and decreases in myelination as shown in animal models and in postmortem analyses from the brains of patients with RLS [28, 29, 127]. There is evidence that the brain iron metabolism disturbances in RLS arise from an increase in the activity of the hypoxia response pathway, specifically hypoxia-inducible factor 1 (HIF-1) [90]. An increase in the activity of HIF also has been identified in patients with OSA as a cellular response to chronic intermittent hypoxia [12, 109]. HIF activation is critical for carotid body-mediated responses to chronic intermittent hypoxia and for hypoxia-related increases in SBP and DBP [91, 109]. HIF-1 consists of two subunits: the oxygen-regulated HIF-1α subunit and the constitutively active HIF-1β subunit. HIF-1α is a transcription factor regulating the expression of the transferrin receptor, transferrin, vascular endothelial growth factor (VEGF), endothelin, and erythropoietin [90, 109]. A postmortem analysis revealed increases in neuronal nitric oxide synthase (nNOS), HIF-1α, and VEGF in the substantia nigra from the brains of patients with RLS versus neurologically healthy controls [90]. HIF-2α (the predominant HIF isoform in endothelial cells) and VEGF also were increased in the brain microvasculature from patients with RLS [90]. The investigators hypothesized that increases in NOS and HIF-1 lead to a decrease in neuronal intracellular iron stores in patients diagnosed with RLS. This NOS-mediated increase in HIF-1 activation may explain alterations in dopaminergic neurotransmission in the brains of patients with RLS [90]. It is unclear, however, whether the postmortem tissue samples from the patients with RLS in the Patton study [90] also had been diagnosed with OSA or other respiratory disorders that may also have led to an increase in HIF expression.

Thus, the HIF pathway may contribute to RLS pathophysiology in different ways: an alteration in HIF-1α expression or function may disrupt the downstream protein expression involved in brain iron metabolism, from effects on sympathetic motor activity through increased carotid body activation, or increased endothelin system activity [12, 85, 90, 91, 109]. Whether NOS-mediated or due to chronic intermittent hypoxia, the interaction between NOS and HIF-1, as well as the other cellular mediators identified by Patton et al. [90], are no doubt complex. Additional studies with greater numbers of patients with RLS are needed to determine whether the HIF pathway contributes to the development of RLS or is a consequence of the disorder.

The increase in NOS activity seen in the post mortem analysis is consistent with genomic analyses in patients with RLS, which identified an association between RLS and *NOS1* gene expression [139]. Variation of the *NOS1* gene would affect the expression of NOS, and in turn the activity of NO. Additional genetic studies identified variants in the genes for *MEIS1* and *BTBD9*, *PTPRD*, and *MAP25K5/SKOR1* [138, 140]. As NO plays a role in the modulation of dopaminergic neurotransmission [139], increased expression of the *NOS1* gene would presumably reduce the effectiveness of dopaminergic neurotransmission, potentially leading to the symptoms of RLS. It would be interesting to determine whether the variants in genetic analysis could be identified in the same post mortem tissue from patients with known RLS in which increases in NOS and HIF expression were observed.

### Hypothalamic-spinal pathways

Clemens et al. [26] attempted to provide a neurologic explanation for the association between RLS and hypertension/CVD. Clemens et al. hypothesized that the clinical aspects of RLS and the association between RLS and CVD, including hypertension, rests on the dopaminergic A11 neurons in the dorsoposterior hypothalamic region, which is distinct from the dopaminergic cells of the substantia nigra. The authors speculated that RLS symptomology initially occurs at night because of the loss of an already low level of dopaminergic availability; any loss of dopaminergic input, either caused by functional or anatomic aberrations, may result in the disinhibition of somatosensory and sympathetic pathways [26]. Normally, there is a balance between serotonin and DA at the spinal cord level. One theory generated by Clemens and Rye and elaborated by Walters and Rye is that a loss of dopaminergic effect at the spinal cord level in RLS leads to enhanced serotonergic drive with resultant increased sympathetic output, vasoconstriction, hypertension, CVD, and stroke [26, 127].

### Cardiac pathways

There are suggestions that PLMS in patients with RLS may be related to changes in cardiac structure. Two recent studies have examined these changes in two different populations of patients [46, 82]. Giannaki et al. examined whether there was further deterioration of left ventricular (LV) structure in patients on dialysis with RLS and PLMS compared to patients without PLMS. Although the changes did not seem of the magnitude to affect function, diastolic LV diameter and mass were significantly increased in the patients with PLMS compared to the controls (*p* = 0.007 and *p* = 0.026, respectively) [46]. Whether the changes in cardiac morphology were due to the uremia or PLMS, however, could not be determined. Mirza et al. observed similar changes increases in LV morphology in patients with RLS and PLMS >35/h. Patients with end-stage renal disease (ESRD) and advanced heart failure were excluded. It is important to note that the patients were referred to PSG for suspected RLS; a diagnosis of the disorder was not objectively obtained. These investigators observed significant increases in LV mass, septal thickness, and systolic and diastolic LV dimensions (*p* < 0.001 for all comparisons) in the patients with >35 PLMS/h versus those with ≤35 PLMS/h. LV ejection fraction was similar between the two groups, indicating that the differences in LV dimensions were not clinically significant. After adjustment (age, sex, other risk factors for hypertrophy), PLMS >35/h was a strong independent predictor of LV hypertrophy severity [OR 2.45 (95 % CI 1.67–1.58); *p* < 0.001] [82]. Both these studies have identified changes in LV morphology that appear not to result in any functional changes in cardiac output. Further study may reveal whether these changes eventually lead to functional cardiac abnormalities in patients with RLS and PLMS or if the reverse is true.

To be sure, little is known about the pathophysiology of RLS and PLMS. However, the above discussion offers plausible explanations as to how RLS/PLMS may influence the development of hypertension and CVD, or the converse. The elevation in CRP observed in the study by Trotti et al., the activation of the HIF pathway reported in the autopsy study by Patton et al., and the results of the studies in patients with gastrointestinal diseases (GI) by Weinstock et al., all propose cellular mechanisms that have been identified to contribute to hypertension, CVD, and stroke and may also be related to the expression of RLS/PLMS [90, 120, 128–131]. More detailed studies examining the possible link between these cellular pathways and RLS/PLMS are needed in order to understand how these processes in RLS also lead to increased risk for hypertension and CVD.

## The effect of comorbidities with RLS/PLMS on hypertension, CVD, and stroke

Adding further to the complexity of the relationship of RLS/PLMS and hypertension and CVD, other comorbidities associated with RLS/PLMS include renal failure, obesity, and iron deficiency—all of which may directly or indirectly predispose patients to CVD. Generally, a higher prevalence of RLS is found in patients with ESRD on dialysis than in the general population (12–60 % vs 7.3 %, respectively) [4, 6, 104, 117]. Lindner et al. [76] observed that patients with ESRD and PLMS have an increased estimated risk of CHD and stroke. Using scores from the Framingham Heart Study to estimate risk, the 10-year estimated risk for stroke or CHD was significantly greater in patients with ESRD and severe PLMS (≥25 PLMS per hour of sleep) versus those with less severe symptoms (*p* = 0.002). Patients receiving dialysis with severe PLMS had a significantly higher risk for CHD compared to patients with milder symptoms (*p* = 0.008) [76].

Several other comorbidities may raise the risk for hypertension and CVD in patients with RLS. Obesity, an established risk factor for hypertension and CVD, has been associated with a significantly higher risk for RLS compared with normal weight controls (OR 1.41; *p* trend <0.0001 for women; OR 1.48; *p* trend = 0.0008 for men) [45]. As noted earlier, OSA is a common comorbidity to RLS/PLMS [87]. Amongst other disease processes, pulmonary hypertension also is a frequent consequence of OSA [114]. A recent study found that approximately 44 % (24/55 patients) of patients with pulmonary hypertension were subsequently diagnosed with RLS; of these, the majority (54 %) reported had moderate to severe symptoms [81]. The average duration of RLS symptoms was 22 months. In addition, many of the patients (42 %) stated their RLS symptoms began after the initiation of treatment for the pulmonary hypertension, suggesting that their symptoms may be treatment induced. Patients with inflammatory GI diseases, such as Crohn’s disease, irritable bowel syndrome, small intestinal bacterial outgrowth, and celiac disease, have a higher risk for RLS than the general population (43, 28, 59, and 35 %, respectively) [128–130]. Many patients with inflammatory GI diseases are iron-deficient—as many as 40 % of patients with celiac disease and active RLS also were iron-deficient [130]—and have high levels of inflammatory markers, such as CRP [15, 112, 124]. This increase in inflammation may begin to explain the relationship between RLS and GI diseases. Also, it is tempting to speculate that the anemia generated by the GI disorders may indirectly contribute to the development of RLS by affecting DA neurotransmission in the brain and spinal cord, resulting in increased sympathetic drive and hypertension. Finally, Angriman et al. [8] have hypothesized that RLS/PLMS when comorbid with attention deficit disorder may increase the risk of CVD through an imbalance in the activity of the autonomic nervous system. These imbalances are thought to be related to sympathetic hyperactivity or imbalances between sympathetic and vagal activity [8]. Although studies examining the processes in support of this hypothesis are needed, it is clear that RLS (and PLMS) is a complex syndrome that can arise through a multitude of pathways.

## Conclusions

The evidence presented in this review argues in support of an association between RLS and PLMS and hypertension and CVD. Support for this hypothesis comes from epidemiologic studies showing an increased prevalence and incidence of hypertension, CVD, and cerebrovascular disease in patients diagnosed with RLS/PLMS. Observational studies provide additional physiologic evidence demonstrating that RLS/PLMS increases several CVD risk factors, such as increased BP and heart rate, and reduced nocturnal BP dipping. A higher incidence of stroke also has been associated with RLS/PLMS. However, as also presented in this review, some studies have presented evidence for an association between RLS/PLMS and CVD or cerebrovascular disease, but that CVD or cerebrovascular disease may increase the risk of RLS. Many of these differences in outcomes may reflect differences in study methodology, subject recruitment, patient population, or in the specific outcomes measured. These discrepancies, perhaps, also attest to the complexity of the relationship between RLS/PLMS and hypertension, CVD, and cerebrovascular disease.

Pharmacologic treatment of RLS/PLMS may reduce the increases in BP and the incidence of vascular complications in patients with RLS/PLMS. DA agonists are considered the first line of pharmacologic therapy for RLS/PLMS [33]. Several studies have documented the reductions in sensory discomfort and improvements in sleep parameters following the administration of DA agonists in patients with RLS/PLMS [34]. Limited evidence shows that treating patients with RLS/PLMS with DA agonists reduces the PLMS-related heart rate response, subsequently normalizing the increases in heart rate [79]. Additional studies are needed to further explore the long-term effects of pharmacologic intervention on CV variables in patients with RLS/PLMS and whether such treatment may reduce the risk of hypertension, CVD, and vascular diseases in these patients.

**Fig. 2:**
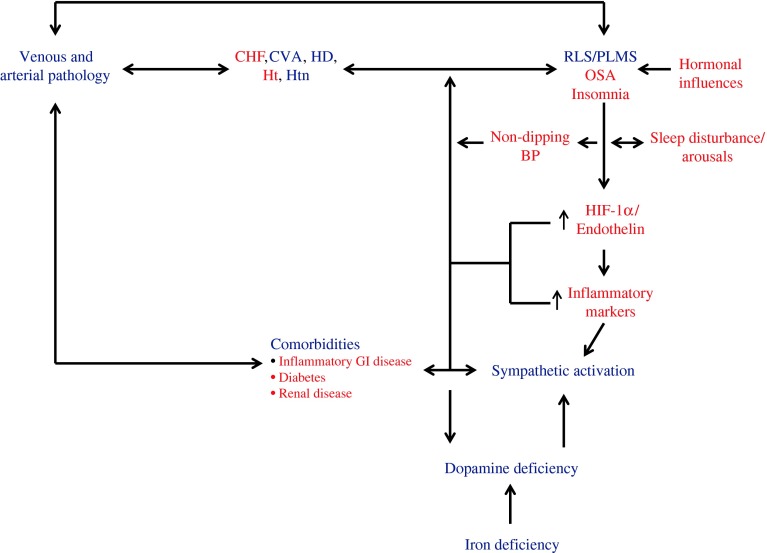
Hypothetical representation of the possible pathways connecting RLS/PLMS, insomnia, and OSA to the development of hypertension and vascular diseases. The original figure, published by Walters and Rye [127], suggested relationships between RLS/PLMS, and hypertension and cardiovascular disease (CVD), and stroke. Labels from the original figure are shown in blue. Red labels represent additional possible relationships between RLS/PLMS, OSA, and insomnia with hypertension and vascular diseases as developed in this review. *CHF* congestive heart failure, *CVA* cerebrovascular accident, *GI* gastrointestinal, *HD* heart disease, *HIF* hypoxia inducible factor, *Ht* heart transplantation, *Htn* hypertension, *OSA* obstructive sleep apnea, *PLMS* periodic leg movements during sleep, *RLS*, restless legs syndrome. Reprinted with permission from Walters and Rye [127]

That RLS/PLMS may be associated with an increase in hypertension, CVD, and vascular diseases is perhaps not surprising. Insomnia and OSA also contribute to an increased risk for hypertension and CVD, albeit likely through different mechanisms. Disorders such as RLS/PLMS, insomnia, and OSA do share commonalities such as reduced sleep and increased arousals during the night, all of which have been shown to increase BP through sympathetic activation and reduce nocturnal BP dipping. Figure 2 is a hypothetical representation summarizing the pathways in which the three sleep disorders, RLS/PLMS, insomnia, and OSA, may contribute to hypertension, CVD, and stroke. The schematic in Fig. 2 attempts to include other factors involved in the expression of RLS such as iron deficiency and renal disease. Although the figure is intended to encapsulate the complexity of the pathways in which RLS/PLMS may contribute to the development of hypertension and vascular diseases, in actuality, many of the connections may be bidirectional. The figure also implies causality between RLS and hypertension and vascular diseases, which has not been scientifically established. However, the variance or the lack of associations in some studies between RLS and hypertension signifies that conclusions regarding this relationship are far from clear.

Understanding the relationship between sleep disorders and hypertension, CVD, and stroke has important clinical implications. The relationship between RLS/PLMS and hypertension may, in part, explain the presence of treatment-resistant hypertension in some patients. Since many patients do not pursue treatment or receive a diagnosis for RLS until symptoms become severe enough to affect their quality of life, the effects of the disease may go unrecognized for an extended time. Thus, the influence of RLS/PLMS and the sleep disruption they cause on heart rate and BP may help to explain why some patients do not achieve therapeutic goals for hypertension. Furthermore, although the increases in BP in many patients with RLS may be small, such increases may be high enough to elevate certain patients into a prehypertension diagnosis, which would result in an increased risk for vascular events. Consequently, polysomnography may be indicated in patients with prehypertension or treatment-resistant hypertension to determine the RLS/PLMS contribution to hypertension. Further research is needed to tease out the complexities of the relationship between RLS/PLMS and hypertension, to determine the effects of pharmacologic therapy on hypertension and related risks, and to bring a greater understanding of the direction of the causality between these two diseases.
